# L’utilisation de l’approche CTC: quel impact sur la couverture vaccinale lors de la campagne préventive de vaccination contre la méningite A avec le MenAfriVac au Togo en 2014?

**DOI:** 10.11604/pamj.2017.27.38.11873

**Published:** 2017-05-12

**Authors:** Dadja Essoya Landoh, Anna-Léa Kahn, Anani Lacle, Kodjovi Adjeoda, Bayaki Saka, Issifou Yaya, Danladi Ibrahim Nassoury, Assima Kalao, Makawa-Sy Makawa, Nsiari-Mueyi Joseph Biey, Andre Bita, Yaovi Temfa Toke, Petit Dörte, Lucile Imboua, Olivier Ronveaux

**Affiliations:** 1 World Health Organization, Country Office, Lomé, Togo; 2 World Health Organization, Headquarters, Genève, Switzerland; 3 Division de l’Immunisation, Ministère de la Santé du Togo, Lomé, Togo; 4 Faculté Mixte de Médecine et de Pharmacie, Université de Lomé, Lomé, Togo; 5 Laboratoire de Santé Publique (EA 3279), Aix-Marseille Université, Marseille, France; 6 Direction Préfectorale de la Santé Golfe, Ministère de la santé, Lomé, Togo; 7 Fonds des Nations Unies pour l’Enfance (UNICEF), Bureau Togo, Lomé, Togo; 8 World Health Organization, IST/WA, Ouagadougou , Burkina Faso

**Keywords:** Meningitis A, MenAfriVac vaccine, CTC, immunization coverage, Togo, Meningitis A, MenAfriVac vaccine, CTC, vaccine coverage, Togo

## Abstract

**Introduction:**

Une campagne de vaccination contre la méningite A avec le vaccin MenAfriVac a été organisée dans les quatre régions septentrionales du Togo du 28 novembre au 07 décembre 2014. L'approche CTC a été utilisée pour la première fois à une grande échelle pour la campagne de vaccination dans dix districts sanitaires du Togo. L'objectif de cette étude était d'estimer la couverture vaccinale et, de déterminer l'effet de l'utilisation de la Chaîne à Température Contrôlée (CTC) sur ces couvertures vaccinales.

**Méthodes:**

L'enquête s'est déroulée du 9 au 14 mars 2015, soit environ 3 mois après la fin de la campagne de vaccination dans ces quatre régions. Le sondage en grappe à deux degrés stratifiés selon les régions a été utilisé. Dans 10 districts, le Togo a fait le choix d'utiliser le vaccin MenAfriVac en CTC.

**Résultats:**

Au total, 2707 ménages ont été enquêtés et 9082 personnes âgées de 1 à 29 ans ont été interviewées. L'âge moyen des personnes enquêtées était de 11,8±7,7 ans et le sex-ratio (H/F) de 1,01. Le nombre moyen de personnes par ménage était de 5,7 et celui des personnes de 1 à 29 ans ciblées par la campagne était de 3,4. Sur les 9082 personnes enquêtées, 8889 (98%) étaient vaccinées. En analyse multivariée, les facteurs associés à la couverture vaccinale avec le MenAfrivac étaient la résidence dans la zone au moment de la campagne (aOR = 4,52 ; 95%IC = [4.07 - 4.97]) et le fait d'être informé de la campagne avant son démarrage (aOR=2,42 ; 95%IC = [2.05 - 2.80]). Par contre, la couverture vaccinale n'était pas différente selon la zone ayant utilisé l'approche CTC ou non (aOR=0,09 ; 95%IC = [-0,27-0,45]). Deux cent sept personnes interrogées (2,3%) ont déclaré avoir eu une Manifestation Adverse Post Immunisation (MAPI) après l'administration du vaccin. Il s'agissait surtout de MAPI mineures à type de fièvre, d'abcès et de gonflement au point d'injection.

**Conclusion:**

Les résultats de cette enquête montrent que l'utilisation de la CTC dans un pays à ressources limitées comme le Togo n'a pas eu un effet négatif sur les couvertures vaccinales. En effet, il n'y avait pas de différence entre la couverture vaccinale dans les zones CTC et celles non CTC. Il importe de capitaliser l'expérience acquise pour l'utilisation des vaccins du Programme Elargi de Vaccination avec l'approche CTC surtout dans les pays à ressources limitées confrontés à la disponibilité de la chaîne de froid.

**Background:**

A vaccination campaign against meningitis A using MenAfriVac vaccine was implemented in the four regions of northern Togo from 28 November to 7 December 2014. CTC approach was first used on a large scale in a vaccination campaign in ten health districts in Togo. This study aims to estimate the immunization coverage and to determine the effect of Controlled Temperature Chain (CTC) approach on these immunization coverages.

## Abstract (en)

**Method:**

We conducted a survey from 9 to 14 March 2015 (for approximately 3 months) after the end of the vaccination campaign in these four regions. Interviewees were selected using two stages cluster sampling stratified according to the regions. MenAfriVac vaccine in Controlled Temperature Chain (CTC) was used in 10 districts, in Togo.

**Results:**

A total of 2707 households were surveyed and 9082 people aged 1-29 years were interviewed. The average age of the individuals surveyed was 11.8±7.7 years and sex-ratio (H/F) was 1.01. The average number of individuals per household was 5.7 and that of persons aged 1-29 years targeted in the campaign was 3.4. Out of 9082 people surveyed 8889 (98%) were vaccinated. Multivariate analysis showed that the factors associated with immunization coverage using MenAfrivac vaccine were: habitual residence in the area at the time of the campaign (AOR = 4.52; 95%CI = [4.07 - 4.97]) and level of information about the campaign before it starts (AOR=2.42; 95%CI = [2.05 - 2.80]). By contrast, there were no differences in vaccination coverage between the areas based on whether the CTC approach was used or not (AOR=0.09; 95%CI = [-0.27 - 0.45]). Two hundred and seven respondents (2.3%) reported that they had Adverse Event Following Immunisation (AEFI) after the administration of the vaccine. These were usually minor AEFI involving fever, abscesses and swelling at the injection site.

**Conclusion:**

Survey results show that the use of CTC in a country with limited resources such as Togo doesn't have a negative impact on immunization coverage. Indeed, there was no difference between immunization coverage in CTC and non-CTC areas. It is important to capitalize on the experience gained in order to use vaccines by Expanded Program of Immunization in CTC approach especially in countries with limited resources in terms of cold chain availability.

## Introduction

Les épidémies de méningites bactériennes constituent un véritable problème de santé publique en Afrique subsaharienne, dans la zone dénommée «ceinture de la méningite» depuis une centaine d'années [1–3]. Cette zone couvre environ 500 millions d'habitants et comprend 26 pays allant du Sénégal à l'Ouest à l'Éthiopie à l'Est. La charge de morbidité liée à la méningite y est très élevée du fait de la méningococcie hyper endémique et d'épidémies aigues, récurrentes et étendues associées à une incidence annuelle pouvant atteindre 1000 cas pour 100 000 habitants. Depuis 2010, un nouveau vaccin dénommé «MenAfriVac™» (vaccin anti méningococcique A conjugué lyophilisé) a été mis au point [4], et introduit en Afrique Subsaharienne, contribuant ainsi à modifier très significativement le profil épidémiologique de ces épidémies [5, 6]. Il est recommandé aux pays surtout situé dans la zone dite ceinture méningitidique à introduire le vaccin MenAfriVac dans leurs programmes nationaux de vaccination. De nos jours, de nombreux vaccins utilisés dans les programmes de vaccination sont en fait plus stables à la chaleur que leur étiquetage actuel ne le laisse entendre [7–11]. Il est souvent extrêmement difficile, voire impossible, de conserver les vaccins dans une chaîne du froid classique, entre 2 et 8°C, surtout dans les pays où les capacités sont limitées pour l'acquisition de la chaîne elle-même comme pour la production d'accumulateurs de froid. De nombreux tests ont donc été faits pour déterminer les vaccins qui peuvent, sur une période limitée de temps et avec un contrôle rigoureux, être gardés en dehors de la chaîne du froid classique [7, 9, 10]. Ces tests ont confirmé qu'il est possible d'utiliser le vaccin MenAfriVac sur une période de quatre jours au maximum à une température pouvant aller jusqu'à 40°C. Ce système de conservation est appelé chaîne à température contrôlée (CTC) [11]. En Octobre 2012, le Contrôleur général des médicaments en Inde a accordé l´autorisation de licence du vaccin MenAfriVac pour une utilisation dans une chaîne de température contrôlée (CTC) à des températures allant jusqu'à 40 °C pour un maximum de quatre jours [11]. Au Togo, près de 2809 cas de méningites ont été enregistrés dans l'ensemble du pays de 2007 à 2012, dont 91 % dans les régions sanitaires Savanes, Kara, Centrale et Plateaux. En Decembre 2014, le Togo a organisé une campagne de vaccination en utilisant le vaccin MenAfriVac dont le but était d'éliminer les épidémies de méningite à méningocoque A, en vaccinant au moins 95% de la population de 1 à 29 ans. Les résultats de cette campagne indiquaient une couverture vaccinale de 102% pour l'ensemble des 4 régions septentrionales du pays [12]. Aussi, le Togo avait fait le choix d'utiliser le vaccin MenAfriVac en CTC dans 10 districts sanitaires. Ces districts ont été choisis sur la base des critères suivants : faible performance de la chaîne du froid; production d'accumulateurs de froid impossible ou limitée; insuffisance des ressources humaines, insuffisance de personnel de santé formé en gestion du PEV; température ambiante maximale inférieure à 40°C [12]. Trois mois après la mise en œuvre de la campagne de vaccination, une enquête de couverture vaccinale a été conduite afin d'évaluer le niveau de réussite de cette campagne et de confirmer les couvertures vaccinales administratives rapportées par les services techniques du ministère de la santé. L'objectif de cette étude était d'estimer la couverture vaccinale dans la population en zone CTC et en zone non CTC après la campagne de vaccination contre la méningite A avec le MenAfriVac.

## Méthodes

**Cadre et période de l'enquête:** L'enquête s'est déroulée du 9 au 14 mars 2015, soit environ 3 mois après la fin de la campagne de vaccination contre la méningite A dans les 4 régions septentrionales du Togo.

**Echantillonnage. Population d'étude:** Il s'agissait des sujets âgés de 1 à 29 ans au moment de la campagne de vaccination. L'enquête a pris en compte à la fois les sujets qui résidaient dans les zones couvertes par la campagne, ainsi que ceux qui étaient absents au moment de la campagne, afin d´obtenir des estimations de la couverture de la population cible ainsi que des estimations de la couverture actuelle. La notion de résidence est déterminée par l'habitation de la personne dans la zone au moins 6 mois avant la campagne ou l'intention d'y rester pendant les 6 prochains mois au moins. La base de sondage est constituée par la liste des villages et cantons avec leurs populations respectives de la Direction Nationale des Statistiques du Togo, établie à partir des données du 4ème recensement général de la population et de l'habitat réalisé en novembre 2010 [13].

**Technique d'échantillonnage:** La méthode d'échantillonnage adoptée pour cette enquête est le sondage en grappe à deux degrés stratifiés selon les régions, qui permet d'estimer la couverture vaccinale à 8% près avec un intervalle de confiance de 95%. Le sondage a été réalisé en prenant en compte le découpage administratif du Togo pour faciliter l'analyse des données et la prise de décision au niveau régional. A l'intérieur de chaque région, un tirage par grappe à deux degrés comme recommandé par l'OMS a été effectué. Au premier degré : les unités primaires de sondage sont constituées des grappes (villages/quartiers) issus des données du 4^ème^recensement général de la population et de l'habitat [13]. La grappe est un espace géographique (village/quartier), sélectionné d'une manière aléatoire, renfermant une population qui contient les ménages à étudier. Dans le cadre de cette enquête, 30 grappes ont été tirées au hasard par zone de sondage constituée de deux à cinq districts sur la base de la liste des villages et cantons avec leurs populations respectives. En plus des 30 grappes, quatre grappes de réserve par zone ont été tirées au hasard en respectant la même procédure, pour d'éventuels remplacements en cas de problèmes d'accessibilité de l'une des 30 grappes tirées. Au total neuf zones de sondage de 30 grappes chacune, couvrant les quatre régions sanitaires ont fait l'objet de l'enquête. Ces neuf zones ont été définies en tenant compte du poids démographique et de la proximité de chaque district au sein de la région. Ainsi, trois zones de sondage de 30 grappes chacune ont été tirées dans la région des Plateaux et deux zones de sondage de 30 chacune ont été tirées dans les trois autres régions. Au deuxième degré : l'unité secondaire de sondage était le ménage vivant dans ces villages/quartiers. Dans chaque ménage tous les sujets âgés de 1 à 29 ans ont été enquêtés. La méthodologie du choix du 1^er^ ménage, détaillée dans le guide de l'enquêteur, permet de réduire au maximum la subjectivité de l'enquêteur. Une fois que le 1er ménage de la grappe est choisi, les autres ménages ont été choisis de proche en proche.

**Collecte des données:** Le questionnaire administré a été adapté du questionnaire qui a été utilisé dans d'autres pays de l'Afrique de l'Ouest (Burkina Faso, Mali). Il était constitué de 3 parties : une partie administrative pour l'identification géographique des ménages; une 2^ème^ partie à l'endroit du chef de ménage et une 3^ème^ partie à l'endroit des personnes ciblées par la campagne. Dans le cadre de cette enquête, tous les chefs-lieux de préfecture et leurs banlieues sont considérés comme milieu urbain. La partie à l'endroit du chef de ménage a permis d'avoir le consentement à l'enquête, le nombre total de personnes dans le ménage, le nombre total de personnes de 1 à 29 ans habitant le ménage. La partie à l'endroit des personnes âgées de 1 à 29 ans a permis de rechercher le statut vaccinal, la notion de survenue de Manifestation Adverse Post Immunisation (MAPI) après l'administration du vaccin.

**Analyse statistique:** L'analyse des données a été réalisée grâce au logiciel Epi Info™ 7. Les variables quantitatives ont été décrites en utilisant la moyenne, et l'écart-type alors que pour les variables qualitatives les proportions ont été calculées. Les estimations de couverture vaccinale et les intervalles de confiance ont été précisés. Notre principale variable d'intérêt était les sujets qui avaient reçu le vaccin MenAfrivac lors de la campagne de vaccination. Le test de Chi 2 ou le test exact de Fisher ont été utilisés le cas échéant en analyse univariée. En analyse multivariée la régression logistique a été réalisée pour identifier les facteurs indépendants associés à la vaccination avec le MenAfrivac au cours de la campagne. Toutes les variables lors de l'analyse univariée avec une valeur de p inférieure à 0,1 ont été inclues dans l´analyse multivariée pour évaluer l'effet ajusté de l'odd ratio (aOR) de chaque variable sur la variable dépendante. Nous avons affecté la valeur “1” à la variable dépendante dichotomique si le sujet avait été vacciné avec le MenAfrivac au cours de la campagne et la valeur “0”dans le cas contraire. Un intervalle de confiance de 95% a été appliqué sur l'ensemble de l'analyse statistique.

**Considérations éthiques:** Un consentement éclairé a été obtenu auprès des participants lors de l'évaluation. Chaque participant était libre de participer ou non à cette enquête. L'anonymat des participants a été respecté et seuls les résultats agrégés font l'objet de cette publication. La campagne de vaccination et l'évaluation ont été approuvées par le Ministère de la Santé du Togo.

## Résultats

**Population d'étude:** au total, 2707 ménages dont la majorité (2426 ménages, 89,6%) en milieu rural ont été enquêtés. Cette enquête a permis d'interviewer 9082 personnes âgées de 1 à 29 ans dont 3509 (36,8%) dans les zones qui ont mis en œuvre la CTC (Tableau 1). La presque totalité des enquêtés (98,6%) ont déclaré qu'ils habitaient dans les quatre régions au moment de la campagne. L'âge moyen des sujets enquêtés était de 11,8±7,7 ans et le sex-ratio (H/F) de 1,01. Au total 714 (7,9%) personnes enquêtées avaient affirmé n'être pas informées de la campagne avant son démarrage (Tableau 1) et 83% des personnes enquêtées connaissaient la maladie contre laquelle elles étaient vaccinées. Les principales sources d'information étaient les crieurs publics/mobilisateurs sociaux (54,0%), suivie par les agents de santé communautaire (12,8%), la radio (12,4%), les enseignants/élèves (12,1%), les agents de santé (9,6%) et les chefs de village (6,1%). Le nombre moyen de personnes par ménage était de 5,7 et celui des sujets de 1 à 29 ans par ménage était de 3,4. Plus du tiers des chefs de ménages n'avaient aucune instruction formelle (Tableau 1). Deux cent sept sujets (2,3%) ont déclaré avoir eu une MAPI après l'administration du vaccin (Tableau 1). Il s'agissait essentiellement des MAPI mineures à type de fièvre, d'abcès et de gonflement au point d'injection. Ces MAPI ont amené les enquêtés à avoir recours surtout à l'automédication dans la majorité des cas.

**Tableau 1:** Caractéristiques de l’échantillon en zone CTC et en zone non CTC, Togo, 2014

Caractéristiques	Total	Mise en œuvre de la CTC		OR	95%CI	p value
		Oui	Non			
**Régions sanitaires**						
CENTRAL	1947 (21,4)	837 (4,.0)	1110 (57,0)	-	-	0,0001
KARA	2375 (26,2)	585 (24,6)	1790 (75,4)			
PLATEAUX	2687 (29,6)	896 (33,3)	1791 (66,7)			
SAVANES	2073 (22,8)	1191 (57,5)	882 (42,5)			
**Enfant vacciné**						
Oui	8885 (98,1)	3419 (97,8)	5466(98,3)	0,78	[0,57 - 1,12]	0,11
Non	170 (1,9)	76 (2,2)	94(1,7)			
**Sexe**						
Masculin	4575 (50,4)	1753 (59,9)	2822 (50,6)	0,98	[0,89 - 1,06]	0,54
Féminin	4507 (49,6)	1756 (50,1)	2751 (49,4)			
**Tranche d'âges**						
≤ 5ans	2267 (25,0)	885 (25,2)	1382 (24,8)	1,02	[0,93 - 1,13]	0,66
> 5ans	6815 (75,0)	2624 (74,8)	4191 (75,2)			
**Informé pour la campagne**						
Oui	8368 (92,1)	3221(91,8)	5147 (92,4)	0,93	[0,79 - 1,08]	0,35
Non	714 (7,9)	288 (8,2)	426 (7,6)			
**Résidant dans la zone lors de la campagne**						
Oui	8957(98,6)	3462(98,7)	5495 (98,6)	1,05	[0,73 - 1,51]	0,88
Non	125 (1,4)	47 (1,3)	78 (1,4)			
**Niveau d’instruction du chef de ménage (N= 2707)**						
*Pas d’éducation formelle*	1052 (38,9)	408 (37,7)	644 (39,6)	-	-	0,17
*Primaire*	1017 (37,6)	432 (40,0)	585 (36,0)			
*Secondaire*	590 (21,8)	225 (20,8)	365 (22,4)			
*Supérieur*	48 (1,8)	16 (1,5)	32 (2,0)			
**Existence de MAPI**						
Oui	207 (2,3)	102 (49,3)	105 (50,7)	0,64	[0,48 - 0,94]	0,002
Non	8741 (97,2)	3306(38,3)	5334 (61,7)			
**Type de MAPI déclarées (N=207)**						
Fièvre	100 (48,3)	58 (56,9)	42 (40,0)	-	-	0,27
Abcès	46 (22,2)	22 (21,6)	24 (22,9)			
Gonflement au point d’injection	28 (13,5)	11 (10,8)	17 (16,2)			
Rougeur	11 (5,3)	4 (3,9)	7 (6,7)			
Céphalées	6 (2,9)	2 (2,0)	4 (3,8)			
Nausées/vomissement	6 (2,9)	1 (1,0)	5 (4,8)			
Prurit	6 (2,9)	2 (2,0)	4 (3,8)			
Autres	4 (1,9)	2 (2,0)	2 (1,9)			

**Estimation de la couverture vaccinale:** Sur les 9082 enquêtés, 8899 (98%) personnes étaient vaccinées au MenAfrivac. Au niveau des zones CTC la proportion des personnes vaccinées était de 97,6% (3426/3509) contre 96, 8% (5398/173) dans les zones non CTC (Tableau 1). Les principales raisons de la non vaccination des 183 personnes étaient l'absence au moment de la campagne (22,6%), la non information de la tenue de la campagne (21,3%), les horaires non adaptées (10,3%) et la peur des effets secondaires (5,2%). Toutes les régions avaient eu une couverture vaccinale supérieure à 97% (Tableau 2). La couverture vaccinale avec carte était de 61,2% au niveau national. Elle était de 51%, 84,1%, 39,5% et 77,9% respectivement dans les régions des Plateaux, Centrale, de la Kara et des Savanes. En analyse univariée, la couverture vaccinale était différente selon les régions (p≤0,001), le fait d'être informé avant le démarrage de la campagne (OR=12,24 ; p≤0,001) et selon la résidence dans la zone au moment de la campagne (OR=103,83 ; p≤0,001) (Tableau 2). Par contre, la couverture vaccinale n'était pas différente selon le sexe (OR=1,12, 95%IC= [0,83 -1,52]), l'âge (OR=0,96 ; 95%IC= [0,68 - 1,36]) et la zone ayant utilisé l'approche CTC ou non (OR=0,75 ; 95%IC= [0,56-1,02]). En analyse multivariée, seules la résidence dans la zone au moment de la campagne (aOR = 4,52 ; 95%IC = [4,07-4,97]) et le fait d'être informé de la campagne avant son démarrage (aOR=2,42 ; 95%IC = [2,05-2,80]) étaient associés au statut vaccinal avec le MenAfrivac (Tableau 3). Par contre l'utilisation de la CTC n'avait pas influencé cette couverture vaccinale (aOR = 0,09 ; 95%IC = [-0.27 - 0.45]).

**Tableau 2:** Estimation de la couverture vaccinale du MenAfrivac après la campagne de masse décembre 2014 au Togo

Caractéristiques	Total	Vacciné au cours de la campagne		OR	95%CI	p value
		Oui	Non			
**Régions sanitaires**						
CENTRAL	1947 (21,44)	1922 (98,7)	25 (1,3)	**-**	**-**	< 0,001
KARA	2375 (26,15)	2340 (98,5)	35 (1,5)			
PLATEAUX	2687 (29,59)	2604 (96,9)	83 (3,1)			
SAVANES	2073 (22,83)	2033 (98,1)	40 (1,9)			
**District ayant utilisé la CTC**						
Oui	3509 (38,6)	3426 (97,6)	83 (2,4)	0,75	[0,56 - 1,02]	0,07
Non	5573 (61,4)	5473 (98,2)	100 (1,8)			
**Sexe**						
Masculin	4575 (50,4)	4488 (98,1)	87 (1,9)	1,12	[0,83 - 1,52]	0,48
Féminin	4507 (49,6)	4411 (97,9)	96 (2,1)			
**Tranche d'âge**						
≤ 5ans	2267 (25,0)	2220 (97,9)	47 (2,1)	0,96	[0,68 - 1,36]	0,88
> 5ans	6815 (75,0)	6679 (98,0)	136 (74,3)			
**Informé pour la campagne**						
Oui	8368 (92,1)	8273 (98,9)	95 (1,1)	12,24	[9,05 - 16,54]	< 0,001
Non	714 (7,9)	626 (87,7)	88 (12,3)			
**Résidence dans la zone de campagne**						
Oui	8957 (98,6)	8845 (98,7)	112 (1,3)	103,83	[69,60 - 154,9]	< 0,001
Non	125 (1,4)	54 (43,2)	71 (56,8)			

**Tableau 3:** Facteurs associés à la couverture vaccinale du MenAfrivac, enquête post campagne au Togo, en analyse multivariée

Caractéristiques	aOR	95%CI	p value
Informer de la tenue de la campagne	2,42	[2,05 – 2,80]	< 0,001
Résidence dans la zone de campagne	4,52	[4,07 – 4,97]	< 0,001
District CTC	0,09	[-0,27 – 0,45]	0,62

## Discussion

L'évaluation de la campagne de vaccination des personnes âgées de 1 à 29 ans a noté une couverture vaccinale, qui était de 98% pour l'ensemble des 4 régions concernées par la campagne, ce qui a dépassé la couverture cible de 95% prévue. Les facteurs associés à cette bonne couverture étaient la résidence dans la zone au moment de la campagne et le fait d'être informé de la campagne avant son démarrage. En dehors de ces deux facteurs associés, la bonne couverture peut être expliquée par i) la perception sociale de la morbidité et de la mortalité de cette méningite; ii) l'efficacité et l'innocuité du vaccin. En effet, seulement 2,3% des personnes interrogées ont déclaré avoir eu une MAPI essentiellement mineures suite au vaccin, et gérées dans la majorité des cas avec succès par les ménages. Cette couverture vaccinale élevée quelle que soient les caractéristiques des populations cibles permet d'espérer une réduction importante de la mortalité et de la morbidité ainsi que la réduction du portage du germe responsable de cette méningite et des épidémies. Les résultats de cette enquête sont à rapprocher de celle réalisée dans les autres pays qui ont déjà bénéficié de cette campagne [5, 6]. Au Burkina Faso, la couverture vaccinale avec le MenAfriVac estimée par enquête dans les 13 régions ciblées par la campagne était de 95,9% [14]. La couverture estimée allait de 90,8% à 98,3% dans les différentes régions ; la couverture estimée était de 97,0% chez les enfants de 2 à 5 ans ; de 97,4% pour les enfants de 6 à 15 ans et de 93,4% pour les personnes de 16 à 30 ans[14]. Tous ces résultats confirment l'engouement des populations des pays de la ceinture méningitique pour la lutte contre les épidémies meurtrières de méningite à méningocoque A. L'introduction de ce vaccin MenAfriVac en Afrique Sub-saharienne, a contribué ainsi à modifier très significativement le profil épidémiologique de ces épidémies [15, 16]. Ceci devraient ouvrir la voie à la mise au point d'autres vaccins conjugués contre d'autres sérotypes de méningocoque, mais aussi d'autres germes responsables de grandes épidémies en Afrique subsaharienne comme le pneumocoque et *Haemophilus influenzae* .

Mais la particularité de cette enquête reste l'efficacité des vaccinations en CTC au même titre que les autres techniques. En effet, l'utilisation de la CTC n'a pas influencé la couverture vaccinale puisqu'il n'y avait pas de différence entre les zones CTC et non CTC. D'autres pays en Afrique subsaharienne ont montré l'efficacité et le bénéfice à utiliser cette technique de vaccination en CTC [17, 18]. Ainsi au Bénin, les vaccinateurs et les superviseurs ont reconnu les avantages évidents de l´approche de la CTC dans les milieux à faible revenu, en particulier dans les zones difficiles à atteindre ou lorsque la chaîne du froid est faible [17]. Le Togo a été l'un des premiers pays où la CTC a été utilisée avec succès à une grande échelle. En effet cette approche a été utilisée dans dix districts sanitaires et a permis de vacciner 1013372 personnes âgées de 1 à 29 ans soit 36,6% des personnes vaccinées lors de cette campagne en 2014 [12]. L´approche de la CTC a le potentiel de réduire la complexité logistique pour maintenir les vaccins dans les températures entre +2 et + 8 °C jusqu'au moment de l´injection du vaccin [11]. Par ailleurs, il n'existe pas de différence entre les MAPI notées avec les vaccinations dans les zones CTC et les vaccinations en zone non CTC [18]. Les résultats de cette enquête montrent ainsi les avantages de la vaccination en CTC dans un pays à ressources limitées comme le Togo. Cette technique pourrait donc être recommandée dans d'autres campagnes de vaccination de masse avec le vaccin MenAfriVac pour protéger toutes les personnes à risque de méningite. Le développement des vaccins pouvant être utilisées avec l'approche CTC serait une avancée très significative comme approche de solution de l'insuffisance de la chaine de froid dans les pays à faible revenue afin d'atteindre et vacciner les populations les plus reculées et d'accès difficile [19], où souvent la chaine de froid est presque inexistante. En effet la nécessité de conserver les vaccins dans une chaîne du froid entre 2° et 8°C est un facteur contraignant pour de nombreux pays [11] dans la mise en œuvre de l'approche atteindre chaque district. La CTC peut alors devenir une alternative lorsque la chaîne du froid ou la production des accumulateurs de froid devient une difficulté contraignante pour les formations sanitaires pour vacciner les populations éloignées [11, 17].

**Limite de l'étude:** les aspects économiques et administratifs de l'approche CTC n'ont pas été pris en compte dans cette évaluation afin de se situer sur l'efficience de cette nouvelle technologie.

## Conclusion

Les résultats de cette enquête montrent que l'utilisation de la CTC dans un pays à ressources limitées comme le Togo n'a pas eu un effet négatif sur les couvertures vaccinales. En effet, il n'y avait pas de différence entre la couverture vaccinale dans les zones CTC et celles non CTC. Il importe de capitaliser l'expérience acquise pour l'utilisation des vaccins du Programme Elargi de Vaccination avec l'approche CTC surtout dans les pays à ressources limitées confrontés à la disponibilité de la chaîne de froid.

### Etat des connaissances actuelles sur le sujet

- Un nouveau vaccin dénommé «MenAfriVacTM» (vaccin anti méningococcique A conjugué lyophilisé) a été mis au point et introduit en Afrique Subsaharienne et a contribué à modifier très significativement le profil épidémiologique des épidémies de méningite à Méningocoque A;
- Nombreux vaccins utilisés dans les programmes actuels de vaccination sont stables à la chaleur;
- Il est possible d'utiliser le vaccin MenAfriVac sur une période de quatre jours au maximum à une température pouvant aller jusqu'à 40°C. Ce système de conservation est appelé chaîne à température contrôlée (CTC).

### Contribution de notre étude à la connaissance

- C'est une perspective pour soutenir le développement des vaccins du Programme Elargi de Vaccination adaptés à l'utilisation avec l'approche CTC;
- L'utilisation des vaccins du Programme Elargi de Vaccination avec l'approche CTC constitue une alternative d'avenir pour faire face aux défis de la disponibilité de la chaîne de froid rencontrés dans les pays pour assurer une couverture vaccinale universelle.

## Conflits d’intérêts

Les auteurs ne déclarent aucun conflit d'intérêts. Dadja Essoya Landoh, Anna-Léa Kahn, Petit Dörte, Lucile Imboua et Olivier Ronveaux travaillent pour l’Organisation Mondiale de la Santé. Les auteurs sont seuls responsables des opinions exprimées dans cette publication et ne représentent pas nécessairement les décisions, les politiques ou les points de vue de l'Organisation mondiale de la Santé.
